# West African Crystalline Maculopathy in Sickle Cell Retinopathy

**DOI:** 10.1155/2015/910713

**Published:** 2015-12-16

**Authors:** Jennifer S. Kung, Theodore Leng

**Affiliations:** Byers Eye Institute at Stanford, Stanford University School of Medicine, Palo Alto, CA 94303, USA

## Abstract

*Purpose*. To describe the first reported case of West African crystalline maculopathy (WACM) from a member of the Benin tribe and explore the association with sickle cell retinopathy.* Methods*. Full ophthalmic examination and high-resolution ocular coherence tomographic imaging.* Patients*. 61-year-old patient from an academic retina practice.* Results*. The patient demonstrated bilateral yellow-green birefringent crystals localized to the inner retina on optical coherence tomography, as well as sickle cell-related neovascularization in the right eye. She reported no consumption of kola nuts.* Conclusions*. Associated retinal vascular disease may be important in the pathogenesis of crystalline maculopathy.

## 1. Case Description

**Figure 1:**
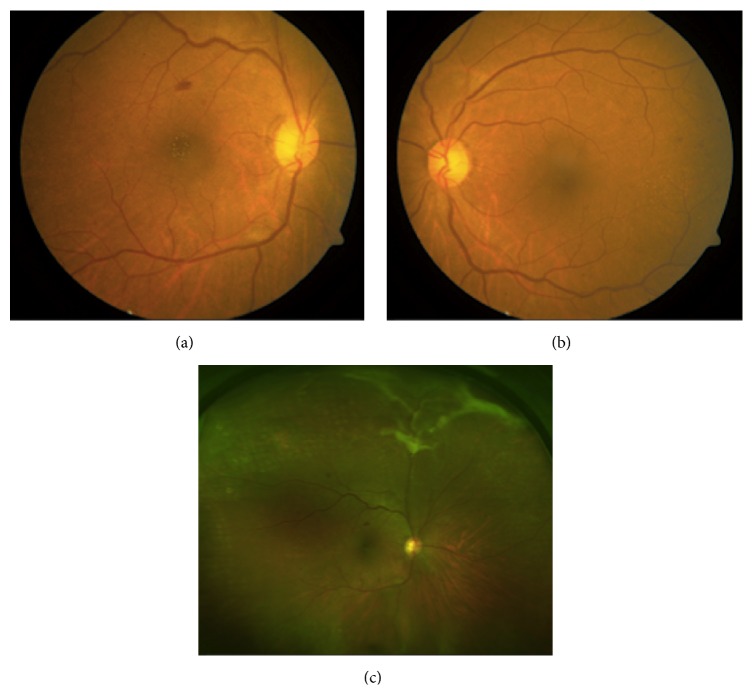
Foveal intraretinal green-yellow crystals were present in the right (a) and left eyes (b) but were asymmetric with relatively more crystals in the right eye. Of note, the right eye also featured regressed sickle cell retinopathy (c) and macular edema, pathologies that were not present in the fellow eye.

**Figure 2:**
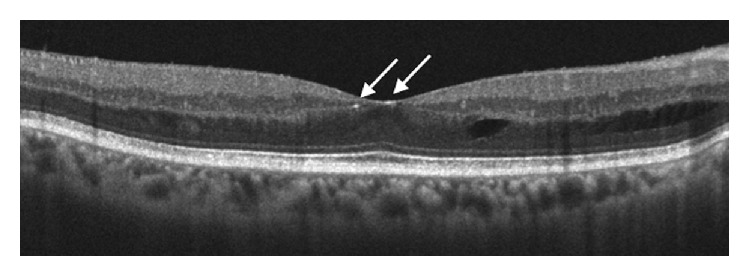
SD-OCT of the right eye demonstrated highly reflective intraretinal crystals in the inner foveal layers (arrows) and outer intraretinal fluid. Other studies have also localized the crystals to Henle's layer [2].

A 61-year-old female with hypertension, diabetes mellitus type II, and sickle cell presented with visual acuity of 20/50 (right eye) and 20/40 (left eye). Dilated fundus examination revealed bilateral foveal refractile yellow-green crystals that were more abundant in the right eye (Figures 1(a) and 1(b)) and regressed sea fan neovascularization in the right superior periphery (Figure 1(c)). Spectral-domain optical coherence tomography (SD-OCT) localized the crystalline deposits to the inner retina (Figure 2). Mild cystoid macular edema was also present in the eye with the crystals, but not the fellow eye. The patient denied prior renal disease, ocular surgery, retinal detachment, use of tamoxifen, canthaxanthine, nitrofurantoin, intravenous drugs, or kola nuts. She had undergone an appendectomy under general anesthesia. The patient was a member of the Benin tribe of Nigeria.

## 2. Discussion

West African crystalline maculopathy was originally described in 2003 by Sarraf et al. in six patients from the Igbo tribe of Southeast Nigeria [1]. Since then, a few other case reports have identified this phenotype in individuals (cumulative number across case reports in parentheses) from other Nigerian tribes (8), Liberia (1), Cameroon (1), Sierra Leone (3), Ghana (4), and one possible case from Egypt (1) [1–5]. All were late middle-aged, generally ranging from 40 to 70 years old and were found to have asymptomatic green-yellow crystals clustered around the fovea without associated retinal vascular or retinal pigment abnormalities. The majority of cases were bilateral but asymmetric. Little is known about the natural history. Crystals may evolve over a period of months [2] and partially reabsorb over several years as well [3]. The severity of the crystalline maculopathy does not appear to correlate with visual acuity [1].

Many theories have been raised regarding the origin of these crystals. A genetic component has been entertained, as this condition is exclusive to West Africans. However, a limited number of eye exams in family members have all been negative. The seminal study implicated West African foods (i.e., kola nuts, cassava, and palm oil) [1], but further studies, including this one, have demonstrated inconsistent consumption. Of interest, many patients have concomitant retinal vascular disease. Of the 14 patients reported by Rajak et al., 11 had diabetes mellitus, 8 had cystoid macular edema, and 2 had sickle cell [2]. Browning described a case series of 3 patients, all of which had diabetes mellitus, and also highlighted that 3 of the 6 patients in the original series had diabetes mellitus [3]. Our patient also had sickle cell disease, with a notable asymmetric presentation of both the sickle cell retinopathy and the crystalline maculopathy predominating in the same eye. Our case supports the hypothesis that associated retinal vascular disease may be critical to pathogenesis of this maculopathy through facilitating the breakdown of the blood-retinal barrier and allowing for the passage of innately or environmentally derived crystal components [3]. The presence of cystoid macular edema in the eye with the crystals also supports a mechanism of blood-retinal barrier breakdown as a potential cause of the crystalline maculopathy. Lastly, while we suspect a vascular cause of blood-retinal barrier disruption in this case, there are other related causes of crystalline maculopathy. Crystals have also been described in unilateral eyes with uveitis, another condition that impacts the blood-retinal barrier [6]. Ultimately, the appearance of crystals could be due to vascular or inflammatory disruption of the blood-retinal barrier. Further long-term studies of patients and their family members would be helpful in elucidating the nature of this maculopathy.
